# Amyloid plaque structure and cell surface interactions of β-amyloid fibrils revealed by electron tomography

**DOI:** 10.1038/srep43577

**Published:** 2017-02-27

**Authors:** Shen Han, Marius Kollmer, Daniel Markx, Stephanie Claus, Paul Walther, Marcus Fändrich

**Affiliations:** 1 Institute of Protein Biochemistry, Ulm University, Helmholtzstr. 8/1, 89081 Ulm, Germany; 2 Central Facility for Electron Microscopy, Ulm University, Albert-Einstein-Allee 11, 89081 Ulm, Germany

## Abstract

The deposition of amyloid fibrils as plaques is a key feature of several neurodegenerative diseases including in particular Alzheimer’s. This disease is characterized, if not provoked, by amyloid aggregates formed from Aβ peptide that deposit inside the brain or are toxic to neuronal cells. We here used scanning transmission electron microscopy (STEM) to determine the fibril network structure and interactions of Aβ fibrils within a cell culture model of Alzheimer’s disease. STEM images taken from the formed Aβ amyloid deposits revealed three main types of fibril network structures, termed amorphous meshwork, fibril bundle and amyloid star. All three were infiltrated by different types of lipid inclusions from small-sized exosome-like structures (50–100 nm diameter) to large-sized extracellular vesicles (up to 300 nm). The fibrils also presented strong interactions with the surrounding cells such that fibril bundles extended into tubular invaginations of the plasma membrane. Amyloid formation in the cell model was previously found to have an intracellular origin and we show here that it functionally destroys the integrity of the intracellular membranes as it leads to lysosomal leakage. These data provide a mechanistic link to explain why intracellular fibril formation is toxic to the cell.

Alzheimer’s disease (AD) is the most common cause of dementia in Western societies^1^. It is characterized by the extracellular deposition of amyloid plaques formed from Aβ peptide and the intracellular deposition of tangles derived from hyperphosphorylated tau protein^2^. While tau neuropathology is usually much better correlated to clinical symptoms and to the progression of disease than Aβ neuropathology^3^, there is strong evidence linking Aβ to AD and suggesting a role as a trigger of disease onset. Aβ pathology, but not tau pathology, is specific for AD^4^. Genetic mutations within Aβ peptide, the amyloid precursor protein (APP) or within the proteins that process APP can lead to familial AD^1^, while no familial AD mutation is known to directly affect tau protein or tau metabolism. Changes in the Aβ-dependent biomarkers but not in the tau-dependent biomarkers can be prognostic for disease development in humans^5^. Injection of fibrillary Aβ(1-42) peptide or Aβ-containing brain homogenates into the brains of tau transgenic mouse models can trigger tau pathology *in vivo*^6^, whereas oligomeric or protofibrillar Aβ intermediates can exhibit neurotoxic properties *in vitro*, alter neuronal plasticity or impair the long-term potentiation in living brain tissue^7^,^8^.

Most previous in-depth biophysical studies on the structure of Aβ peptide focused on the molecular conformations of the peptide in the fibrils or in prefibrillar aggregates but could not investigate the natural context of the aggregates in a deposit. Analysis of Aβ states formed *in vitro* with techniques such as solid-state NMR spectroscopy, hydrogen exchange and cryo electron microscopy, revealed the global topology and peptide dimer structure of the peptide in the fibril or the residue-specific β-sheet conformation^9^,^10^,^11^,^12^,^13^,^14^. The three-dimensional (3D) organization of the fibrils in a deposit remained largely elusive, but based on techniques such as conventional transmission electron microscopy (TEM); that is without using tomographic methods, or light microscopy star-like plaque structures were proposed^15^. For example, observation of a Maltese cross in the polarizing microscope is commonly interpreted to originate from fibrils that radiate out from a center^16^. The plaque structures formed by Aβ peptide in the course of AD are characteristic for the disease and different from the kind of deposits formed from the peptide in non-demented elderly individuals^4^. Furthermore, the environment of Aβ fibrils inside the brain is evidently more complex than in a test tube as *in vivo* amyloid deposits comprise, for example, glycosaminoglycans (GAGs) or lipids^17^,^18^,^19^.

In the present work we have used electron tomography to analyze the 3D arrangement of fibrils and of fibril-cell interactions within amyloid plaques formed from Aβ peptide. The plaques were obtained from a cell culture model that exposes monocytes or other cells to extracellular Aβ peptide^20^,^21^. The cell system remodels the natural situations of astrocytes, microglia and other cells being exposed to extracellular Aβ peptide in the brain^22^. One form of the cell model involves human monocytic THP-1 cells that internalize the peptide and mediate its conversion into amyloid plaques (Fig. 1a). Fibril formation was previously found to be toxic^20^,^21^, and there was evidence that fibril formation starts within an endocytic compartment^19^. The formed amyloid showed classical features of amyloid formed *in vivo*, such as an association with GAGs and lipids^20^,^21^, and a growth kinetics resembling the growth of plaques within the brains of murine models of AD^23^,^24^ and the nucleated polymerization kinetics seen *in vitro*^25^.

## Results

### Formation of Aβ amyloid plaques in cell culture

**Figure 1:**
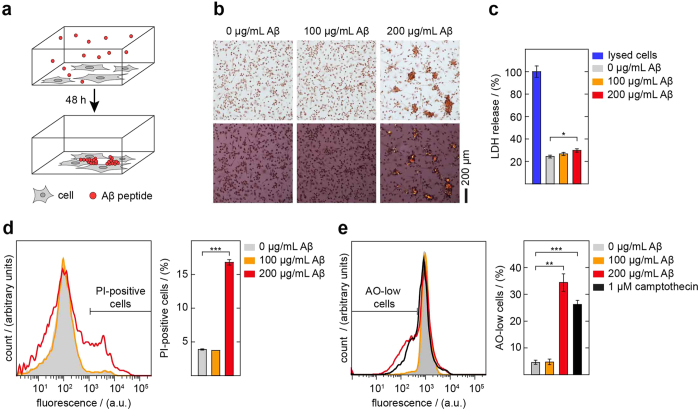
Generation of Aβ plaque formation is toxic to the cells. (**a**) Schematic representation of the cell model of Aβ plaque formation. Amyloid deposits accumulate with 200 μg/mL Aβ over a period of 48 h. (**b**) Bright field (top) and dark field (bottom) polarizing microscopy images of CR-stained samples in which cells were incubated without or with different concentrations of Aβ peptide for 48 h. (**c**) LDH release assay to measure cellular viability after a 48 h incubation period or without Aβ peptide as indicated. The LDH release of cells that were incubated with Cell Lysis Solution (Bio Vision) for 30 min prior to the measurement was set to 100% (n = 3; mean ± SD). (**d**) Flow cytometric analysis of PI-stained cells after incubation with or without Aβ as indicated for 48 h. Left: Raw flow cytometry data. Right: Quantification of PI-positive cells (n = 2; mean ± SD). (**e**) Flow cytometric analysis of AO-treated cells after incubation with or without of Aβ peptide for 48 h or with camptothecin for 4 h. Left: Raw flow cytometry data. Right: Quantification of AO-low cells (n = 3; mean ± SD).

The amyloid plaques formed by the cell model stain with the amyloid-binding dye Congo red (CR) and show CR green birefringence in the polarizing microscope (Fig. 1b), the gold standard of amyloid detection in pathological diagnosis^26^. The amount of amyloid formed in a well depended on the concentration of Aβ peptide that was added to the supernatant (Fig. 1b). Under the presently used set of conditions we found CR green birefringent amyloid deposits if we incubated THP-1 cells for 48 h with 200 μg/mL Aβ, whereas 100 μg/mL Aβ did not produce discernible amyloid deposits as judged by CR green birefringence (Fig. 1b). Amyloid plaque formation was toxic to the cells, as demonstrated by the release of lactate dehydrogenase (LDH) (Fig. 1c), which depends on the perforation of the plasma membrane and the release of large-sized LDH protein into the medium. Incubation of the cells with 200 μg/mL Aβ peptide led to significant levels of toxicity under the conditions of our experiment, while there were only mild, if any effects with 100 μg/mL Aβ (Fig. 1c). The extent of toxicity seen with 200 μg/mL Aβ was nevertheless much lower than the toxicity seen with Cell Lysis Solution (BioVision), which led to an almost complete lysis of the cells (Fig. 1c, blue bar).

The membrane damage was confirmed by propidium iodide (PI) staining, which uses a relatively small molecule to measure the perforation of the plasma membrane, and acridine orange (AO) staining, which measures lysosomal leakage. Staining of the cells exposed to 200 μg/mL Aβ with the DNA-intercalating dye PI led to a significant labeling the cells. By contrast, cells exposed to only 100 μg/mL peptide did not give a strong PI signal (Fig. 1d), which is consistent with the above LDH assay in showing that the lower Aβ concentration did not suffice to provoke significant membrane damage. We then tested staining of the cells with AO, a dye which exhibits a pH-dependent shift and reduction of the fluorescence emission signal as it exits from acidic vesicles into the cytoplasm. We found a quenched fluorescence signal if cells were exposed to 200 μg/mL Aβ peptide (Fig. 1e), which was similar to the fluorescence reduction seen if cells were treated with camptothecin, a widely known inducer of apoptosis and lysosomal leakage^27^. These data imply that the cell death follows fibril formation and involves the perforation of cellular membranes as well as a leakage of lysosomal cargo.

### Superstructure of an amyloid deposit revealed by scanning electron transmission tomography

We embedded samples of the amyloid forming cells into epoxy resin and focused on cells that were exposed to 100 μg/mL Aβ to increase the chance of observing living/intact cells. We cut out 700-nm thick sections from the embedded cells in a plane parallel to the substrate and subjected them to scanning electron transmission microscopy (STEM). Contrary to the CR data presented above, STEM revealed significant amounts of extracellular amyloid fibrils in this sample. These observations demonstrate that 100 μg/mL Aβ was sufficient to induce fibrillogenesis and suggested further that the quantity of the deposited filaments may not have been substantial enough to become detectable by CR (Fig. 1b).

**Figure 2:**
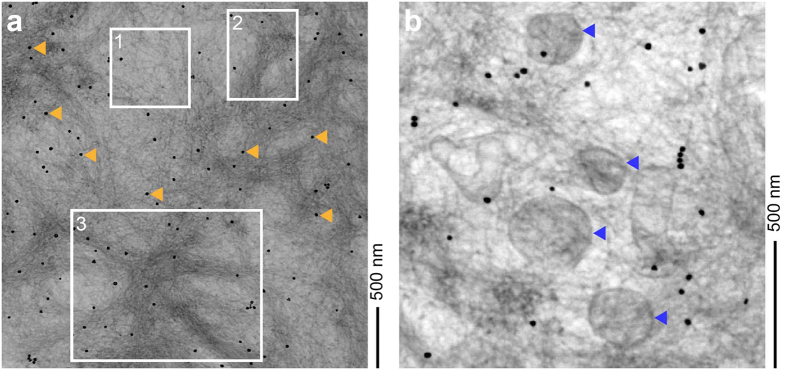
STEM micrographs showing the fibril network structure of an Aβ amyloid plaque. (**a**) STEM micrograph of a 700 nm section of an Aβ amyloid plaque, showing different network structures: fibril meshwork (1), fibril bundle (2) and amyloid star (3). Orange arrowheads indicate gold particles which were used for the image alignment of the tomograms. Scale bar: 500 nm. (**b**) STEM micrograph of a 700 nm section showing vesicular inclusions (blue arrowheads).

The obtained STEM images revealed three different types of fibril network structures that we term here meshwork, fibril bundle and amyloid star. All three structures coincided within the same overall deposit and with no discernible borders (Fig. 2a). An amyloid star consisted of fibrils that radiated out in different x/y-directions. A fibril meshwork consisted of filaments that did not present any obvious overall orientation, whereas a fibril bundle contained filaments, which were aligned in parallel to one another. The fibril width distribution was indistinguishable in the three network structures and the average width values were 8.9 ± 1.5 nm for fibril meshwork, 8.4 ± 2.0 nm for the bundle and 8.7 ± 1.4 nm for an amyloid star. Hence, the different network assemblies are constructed from morphologically indistinguishable fibrils. This conclusion is consistent to a previous tomographic study on the arrangement of fibrils in a non-cerebral amyloid disease where there was also evidence for meshwork, bundle and star-like assemblies^28^, suggesting their relevance for different forms of amyloidosis.

### Tomographic views of the interactions with lipid inclusions and cell surfaces

**Figure 3:**
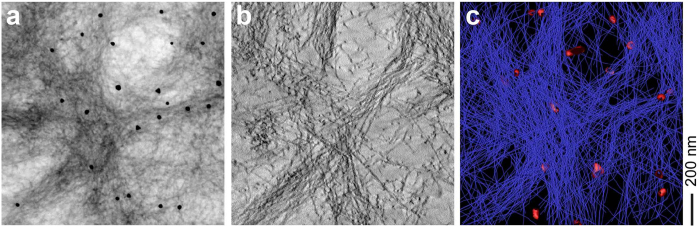
Electron tomogram of an amyloid star. (**a**) Two-dimensional projection of a 700-nm section cut through an amyloid star. (**b**) Virtual section through the tomogram (z-axis thickness: 216 nm). (**c**) 3D-model of the fibril network in an amyloid star. Fibrils are shown in blue, lipid vesicles are shown in red.

**Figure 4:**
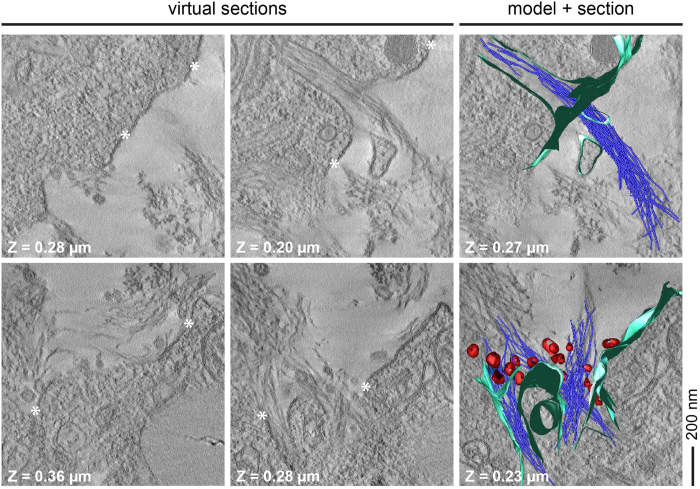
Visualization of Aβ fibril-cell membrane interaction by STEM tomography. Left and middle column: Virtual sections through the tomogram of cells incubated with 100 μg/mL Aβ for 48 h at different z-axes positions as indicated. Asterisks denote the lipid bilayer of the cell. Right column: Overlay of the 3D model section. Fibrils are shown in blue. Cell membrane is highlighted in green. Lipid vesicles are shown in red.

We further noted that the fibril deposits contained significant quantities of vesicular lipid inclusions that varied in diameter from approximately 50 to 300 nm (Fig. 2b). These inclusions were seen in all three network structures. Based on a tilt angle image series we constructed STEM tomograms at a resolution of ≤5 nm that could resolve, for example, the cellular lipid bilayer structure (see below), which is known to be ~5 nm across. In the tomogram of an amyloid star we could trace the fibrils through consecutive virtual sections to generate a 3D-model (Fig. 3c). There was no evidence for strong interactions between fibrils and the lipid vesicles. Focusing on the areas of the amyloid deposits where the fibrils were in close vicinity to the cells we found bundles of fibrils penetrating into tubular invaginations of the plasma membrane (Fig. 4). These bundles occasionally contained a corona of small lipid vesicles that varied in diameter from approximately 50 to 100 nm, corresponding to the known size rage of exosomes (40 to 100 nm)^29^. Exosomes are lipid vesicles that are released from the cells by exocytosis and that are known to be associated with protein aggregates in AD other neurodegenerative diseases^30^,^31^. While our STEM tomograms could resolve the lipid bilayer structure of the plasma membrane (Fig. 4, asterisk), it was sometimes difficult to track the membranes through consecutive virtual sections of the tomogram. This problem was particularly severe along the z-axis direction due to the missing wedge effect^32^ and it was not possible to determine whether there is any direct effect or impairment of the fibrils on the membrane integrity at this site.

## Discussion

Electron tomography is an increasingly powerful technique to analyze the 3D structure of cells and biomolecular assemblies, which was previously used, for example, to illuminate the 3D structure of individual prion or amyloid fibril aggregates^33^,^34^, the interactions between fibrils and lipid vesicles^28^,^35^ and the assembly of fibrils within the amyloid deposits formed from serum amyloid A protein from systemic AA amyloidosis^28^. In the present study it enabled us to determine the 3D fibril assembly within an amyloid deposit from Aβ peptide. We find that the deposits consist of three main types of network structures, termed meshwork, fibril bundle and amyloid star (Fig. 2a). In particular star-like arrangements have redundantly been implied for the structure of Aβ amyloid deposits in the brain tissue of patients and AD animal models^15^,^36^. While these studies demonstrate the relevance of star-like assemblies for amyloid formation *in vivo*, previous pictures were based on light microscopy or conventional TEM methods without tomography. Based on the present study, we can now provide a 3D view of an amyloid star that depicts the relative assembly of individual filaments (Fig. 3c).

The recorded tomograms further revealed the interaction between fibrils and differently sized lipid structures. The presence of lipids in Aβ plaques was previously demonstrated with techniques, such as Fourier-transform infrared or coherent anti-Stokes Raman scattering microscopy^37^, but it was difficult to establish with these previous techniques the physical nature of the observed lipid assemblies or to identify the mode of their interactions with the fibrils. Using STEM tomography, both questions could now be addressed.

We could reveal, on the one hand, that the deposit-associated lipids occur in the form of extracellular lipid vesicles and we could detect both small-sized (diameter of ≤100 nm) as well as large-sized lipid inclusion with dimeters of up to 300 nm. While the larger lipid structures could be remnants of dead cell bodies, small size-vesicles appear to be exosomes that are known to be relevant in the etiology and spreading of AD and other neurodegenerative diseases^30^,^31^. Exosomes are actively released from living cells by exocytosis and the observation of exosome-like vesicles as a corona around fibril bundles that protruded from tubular invaginations of the plasma membrane (Fig. 4) suggested that fibrils and exosomes may have encountered in the exocytic pathway such that they became jointly exported from the cells. Indeed, there has previously been evidence that intracellular fibril bundles are particularly prominent in multivesicular bodies^21^, and this mechanism would reconcile our previous observations of an intracellular origin of fibril formation in our cell model^21^ with the fact that amyloid deposits are usually seen in the extracellular space^26^. However, it is not currently possible to discriminate whether the fibril bundles seen at the cell surface arise from attempts of the cells to phagocytose and degrade the extracellular filaments or from their exocytosis after intracellular fibril nucleation. Both possibilities could be supported by available literature evidence^38^,^39^.

On the other hand, our tomograms did not show any strong interactions between the lipids and the filaments. This observation implies that the detected lipid inclusions were mainly captured by the porous sieve structure of the fibril network rather than attracted by strong complementary chemical forces.

The obtained data ultimately provide a mechanistic link between the intracellular fibrils formation and cellular toxicity. It was known from previous studies that fibril formation is toxic to the amyloid forming cells and that it precedes fibril formation^20^,^21^. In addition, it was found that intracellular fibrils deform the structure of intracellular lipid vesicles and prick through the vesicular membrane into the cytoplasm^21^. However, it was not clear whether there was any direct link between these events and the encountered cellular toxicity as lysosomal leakage could not previously be demonstrated. Lysosomal leakage is known to lead to cellular toxicity^40^. With our AO assay we can now show the functional disruption of the intracellular membranes and lysosomal leakage in these amyloid-producing cells (Fig. 1e). Taken together with current PI and LDH data there is strong support to a perturbation of the cellular membrane integrity provoked by the formation of amyloid fibrils.

It is possible that further damage occurs between fibrils and the plasma membrane at the cell surface. The tomograms recorded with amyloid bundles deterring the cell surface structure imply at least strong effects of the filaments of the cell border. From our tomograms, however, it is not clear whether or not these interactions destroy the membrane integrity of the tubular invaginations or not. Nor can we exclude an additional contribution from amyloid intermediates on the cellular toxicity, such as oligomers or protofibrils, that were clearly shown to be toxic to cells in numerous experimental settings^7^,^8^. Nevertheless, it is clear from the obtained data that fibril formation is not beneficial to the affected cells and causes significant toxicity. This conclusion is consistent with histological observations that amyloid plaques within the brain are typically surrounded by halos of altered neuronal activity^41^.

## Material and Methods

### Source and preparation of Aβ peptide

Wild type Aβ(1-40) peptide was recombinantly expressed in *E. coli* as a fusion protein that was cleaved off during purification. The native peptide without any remaining tags or extra residues was purified by liquid chromatography as described previously^42^. The amino acid sequence of the purified peptide was confirmed by mass spectrometry and contained no posttranslational modifications.

### Generation of Aβ amyloid plaques in cell culture

THP-1 cells were plated out at a density of 3·10^5^ cells/mL in a clear 96-well plate (Greiner) that was either supplemented with sapphire discs (STEM tomography) or coverslips (light microscopy). The cells were differentiated in RPMI 1640 medium (GE healthcare) supplemented with 10% (v/v) heat-inactivated fetal bovine serum (Gibco), 1% (v/v) Antibiotic-Antimycotic solution (Gibco) and 50 ng/mL phorbol 12-myristate 13-acetate (PMA, Sigma) at 37 °C and 5% CO_2_. After 48 h cells were incubated in PMA-free medium for 6–8 h before the medium was replaced again with PMA-free medium containing 100 or 200 μg/mL Aβ(1-40) and further incubation for 48 h. In a next step, cells grown on a coverslip were stained with CR and analyzed by light microscopy or cells grown on sapphire discs were embedded in epoxy resin and prepared for STEM tomography.

### CR-staining of cell culture-derived amyloid plaques

Samples grown on a coverslip were washed once in 100 μL phosphate-buffered saline (PBS, 137 mM sodium chloride, 2.7 mM potassium chloride, 8 mM di-sodium hydrogen phosphate, 2 mM potassium dihydrogen phosphate, pH 7.4) and fixed in 100 μL ice-cold methanol for 10 min at 4 °C. The methanol was removed and the samples were incubated for 45 min in 100 μL CR solution [0.6% CR (w/v), 80% ethanol (v/v), 3% sodium chloride (w/v)] on an orbital shaker (Heidolph) at a speed of 80 rpm. The samples were washed three times in 100 μL H_2_O and incubated for 2 min in Mayer’s hemalum solution (Roth). The unbound hemalaun was removed by washing the samples once in 70% (v/v) ethanol and three times in 100 μL H_2_O. The coverslip was carefully removed from the 96-well plate and dehydrated by plunging it in 90% (v/v) ethanol, 100% (v/v) ethanol and two times in 100% (v/v) xylol, respectively. Residual liquid after each plunge was removed with a filter paper. The coverslip containing the dehydrated samples was mounted with a drop of Roti-Histokitt (Roth) on a glass slide and analyzed with a polarizing light microscope (Nikon Eclipse 80i).

### Preparation of specimens in epoxy resin for transmission EM

The sapphire discs were removed from the 96-well plate and dipped once in 95% (v/v) 1-hexadecene (Sigma-Aldrich). Two sapphire discs oriented face-to-face and separated by a gold ring (3.05 mm diameter, 2 mm central bore; Plano) were mounted into a holder (Engineering Office, M. Wohlwend) and placed into a Wohlwend HPF Compact 01 high-pressure freezer (Engineering Office, M. Wohlwend). The samples were pressure-freezed with 2100 bar. After high-pressure freezing, the sapphire discs were separately incubated in 1.5 mL precooled (−87 °C) sample tubes filled with 1 mL freeze substitution solution [0.2% (w/v) osmium tetroxide, 0.1% (w/v) uranyl acetate, 5% (v/v) distilled water in acetone]. After the tube had been warmed up to 0 °C within 17–22 h the samples were washed three times with 1 mL 100% (v/v) acetone before incubating the sample in 1 mL epoxy resin solution with increasing concentrations [25%, 50% and 75% (v/v)] for 1 h at room temperature each. Finally, the samples were incubated in 100% (v/v) epoxy resin solution over night at room temperature. The sapphire discs were transferred into a clean tube containing 0.25 mL 100% (v/v) epoxy resin and incubated for 24 h at 60 °C to polymerize the resin. After polymerization the sample blocks were stored at room temperature.

### STEM and STEM tomography

Specimen preparation was mainly carried out as described before^28^. In brief, 700 nm-thick sections were cut off from the epoxy resin block parallel to the plane of the sapphire disc with an Ultracut UCT ultramicrotome (Leica) equipped with a diamond knife (Diatome). Before mounting the slice onto a 300 mesh copper grid, the grid was plasma-cleaned with an Edwards plasma cleaning system and dried for 10 min at room temperature. Afterwards, a droplet of 10% (w/v) poly-L-lysine (Sigma Aldrich) in water was added on the sample and dried for 5 min at 37 °C. The grid with the slice on it was treated on both sites with 15 μl of a solution containing 25 nm gold particles (Aurion) diluted 1:1 with water. Finally, the grid was coated on both sites with a 5 nm carbon layer using a BAF 300 electron beam evaporation device (Balzers). Images were recorded in the STEM mode with a Jeol JEM-2100F (Jeol) with an acceleration voltage of 200 kV and a bright field detector (Jeol) at a size of 1,024 × 1,024 pixels. Images were recorded at different tilt angles ranging from −70° to +70° with a tilt increment of 1.5°. The complete tilt series contained 94 individual images. Each image was acquired with an exposure time of 22 s. To generate a 3D model of the individual images, the images were first aligned to an image stack and subsequently reconstructed computationally using a weighted back-projection algorithm. The final generation of the 3D model was done by tracing the fibrils and lipid vesicles manually within different virtual sections of the tomogram. The reconstruction and 3D modeling was carried out using the IMOD software package^43^ version 4.7.12.

### LDH release assay of cellular viability

Cell viability was determined by an LDH-Cytotoxicity Colorimetric Assay Kit II (BioVision) according to manufacturer’s instructions. In brief, differentiated THP-1 cells were incubated with 100 or 200 μg/mL Aβ peptide for 48 h (see above). To obtain 100% LDH release the cells were incubated with 10 μL cell lysis solution at 37 °C 30 min prior to the measurement. 10 μL of the cell culture supernatant or 11 μL of the supernatant from lysed cells were transferred into a new clear 96-well plate (Greiner) and incubated with 100 μL LDH reaction mix for 30 min in the dark at room temperature and 80 rpm on a horizontal platform shaker (Heidolph). The absorbance of all samples was measured with a 96-well plate reader FluoStar Omega (BMG Labtech) at 450 nm and 650 nm. For data analysis, the absorbance at 650 nm was substracted from the absorbance at 450 nm.

### PI assay of cellular viability

THP-1 cells were plated out at a density of 3·10^5^ cells/mL in a clear 24-well plate (Greiner), differentiated and incubated with Aβ peptide to generate amyloid plaques as described before (see above). Cells were washed once in PBS, pH 7.4 and incubated with 200 μL trypsin/ethylendiaminetetraacetic acid solution for 5 min at 37 °C. The reaction was stopped by adding 500 μL FBS-containing RPMI 1640 medium. Differentiated THP-1 cells were detached from the plastic surfaces by carefully tapping the plate onto the bench and gentle scratching with a cell scraper and transferred into a flow cytometry tube. Remaining cells in the culture dish were recovered by washing the well once with 200 μL PBS, pH 7.4. Cells were centrifuged for 5 min at 100·g and the pellet was washed twice in 500 μL PBS, pH 7.4. Finally, the cells were resuspended in 100 μL FACS-buffer (PBS containing 0.5% (w/v) bovine serum albumin, 0.1% (w/v) sodium azide) and incubated for 1 min at room temperature with 10 μL PI stock solution (10 μg/mL in PBS, pH 7.4) in the dark and analyzed by a FACSVerse flow cytometer (BD Biosciences) at an excitation wavelength of 488 nm and an emission wavelength of 586 nm ± 42 nm. We measured 10,000 events.

### AO assay of lysosomal leakage

THP-1 cells were plated out at a density of 3·10^5^ cells/mL in a clear 24-well plate (Greiner), differentiated and incubated with Aβ peptide to generate amyloid plaques as described above. As a control, a set of cells was treated with 1 μM camptothecin (Calbiochem) for 4 h to induce apoptosis and lysosomal leakage. Then, 5 μL AO of a 500 μg/mL stock solution (Sigma-Aldrich) were directly added to the cell culture medium of the cells and incubated for 15 min at 37 °C. Afterwards, the medium was collected in a flow cytometry tube and cells were detached from the plastic surface by incubation with 200 μL trypsin/ethylendiaminetetraacetic acid solution for 5 min at 37 °C. The reaction was stopped by adding 500 μL FBS-containing RPMI 1640 medium, cells were pipetted up and down for three times and transferred into a flow cytometry tube. The well was washed once with 1 mL PBS, pH 7.4 and the remaining cells were collected in the same flow cytometry tube. The tubes were then centrifuged at 400·g for 5 min, the supernatant was discarded and the pellet was resuspended in 200 μL PBS, pH 7.4. AO fluorescence was analyzed by a FACSVerse flow cytometer (BD Biosciences) at an excitation wavelength of 488 nm and an emission wavelength of 700 nm ± 27 nm. We measured 10,000 events.

## Additional Information

**How to cite this article:** Han, S. *et al*. Amyloid plaque structure and cell surface interactions of β-amyloid fibrils revealed by electron tomography. *Sci. Rep.* **7**, 43577; doi: 10.1038/srep43577 (2017).

**Publisher's note:** Springer Nature remains neutral with regard to jurisdictional claims in published maps and institutional affiliations.
